# Factors Influencing Response Rates in National Internet-Based Questionnaires: Analysis of a Large National Survey

**DOI:** 10.2196/89761

**Published:** 2026-09-30

**Authors:** Søren Birkeland, Anders Haakonsson, Sören Möller

**Affiliations:** 1 Odense University Hospital, Open Patient data Exploratory Network (OPEN), J. B. Winsløws Vej 21, 3rd floor, 5000 Odense, Denmark, 45 5142 1413; 2 Forensic Mental Health Research Unit Middelfart (RFM), Department of Regional Health Research, University of Southern Denmark, Middelfart, Region of Southern Denmark, Denmark; 3 Department of Public Health, University of Southern Denmark, Odense, South Denmark, Denmark

**Keywords:** multidisciplinary research, public health, social research, survey methods, survey participation

## Abstract

**Background:**

Digital mailbox platforms established by authorities for secure communication with the general populace are increasingly available for distribution of surveys. While such solutions constitute an attractive way of easily distributing surveys, nonresponse remains a critical problem, warranting measures to increase participation.

**Objective:**

We aimed to investigate questionnaire characteristics predicting participation and the effect of reminders among men in a survey distributed through a national digital mailbox solution.

**Methods:**

This study used an internet-based survey among men aged 45 to 70 years; the survey was sent out in 2 waves through a digital mailbox used by Danish authorities. Predictors for response were analyzed using logistic regression.

**Results:**

Of 24,000 invitations, 22,288 participants accepted, with 6756 (30.3%) completing the survey. We found some evidence suggesting a rule of thumb that reminders may add another half to the response rate achieved in a preceding invitation, with 21% and 10% of responses obtained before and after the reminder, respectively (in the first wave). Longer questionnaire variants (odds ratio [OR] 0.77, 95% CI 0.66‐0.88; *P*<.001 [longest vs shortest variants]), questionnaires with attached informational material (OR 0.92, 95% CI 0.85‐0.98; *P*=.02[with vs without material]), or with potentially distressful emotional content (OR 0.91, 95% CI 0.85‐0.98; *P*=.008 [most vs least distressful content]) tended to yield lower response rates. Furthermore, older age was associated with a higher response rate when comparing the oldest (65‐70 years) to the youngest (45‐55 years) age groups (OR 2.49, 95% CI 2.31‐2.70; *P*<.001).

**Conclusions:**

We confirmed some previous findings obtained with other modes of survey distribution on the association between response rates and questionnaire characteristics, showing that it is important to pay attention to specific characteristics when designing internet-based surveys distributed through digital mailboxes. Nonresponse may constitute a considerable and increasing challenge, and reminders may help substantially increase participation.

## Introduction

In many countries, electronic survey distribution through internet-based services and web platforms is a relatively new opportunity for research, and it is being increasingly used for gathering data to inform policy and practice [1]. The Danish digital mailbox (e-Boks) is one example [1], with similar digital mailbox solutions available in a rising number of countries (Norway, Sweden, Greenland, and Ireland) [2]. Survey delivery through this channel has provided many new advantages and opportunities for easy survey distribution and straightforward subsequent analysis of the gathered electronic data. Furthermore, a digital mailbox with web access constitutes an easy platform for potential respondents to access and fill out surveys without requiring, for example, manually filling out and posting paper material; therefore, it could be hypothesized that such platforms would promote research participation. Despite these advantages, nonparticipation remains a critical challenge for internet-based surveys, including those distributed via digital mailboxes, with potential implications for practice, policymaking, and public opinion [1]. Moreover, there is significant evidence from multiple studies that participation rates in public health surveys have been generally decreasing for the last several decades [3,4]. Correspondingly, there is emerging evidence that participation in web-based health-related surveys is declining [5-7]. Various measures have been proposed to mitigate this, such as use of monetary incentives to increase response rates [6]. Another traditional approach is to use reminders [7,8].

In parallel, previous research has demonstrated that participation varies with geography, with participation usually being higher in less-populated areas [9-13]; participation also usually increases with older age, although this tendency can change when older people are less familiar with, for example, digital platforms [5-7,13-17].

Participation may, however, also be dependent on features of the survey [18]. Most of our knowledge on this point derives from surveys from the predigital era. Hence, limited research exists on internet-based solutions and, particularly, on surveys distributed through national-level web platforms. This paper reports on experiences from a research project using a national authority’s digital mailbox solution, focusing on survey characteristics that predict response rates and the effect of reminders.

## Methods

### Overview

This is a secondary analysis of data obtained from a large, cross-sectional, national-level web survey about health care user involvement in decision-making regarding prostate-specific antigen (PSA) testing; the survey was distributed among adult men aged 45 to 70 years; this age range was chosen based on US Preventive Services Task Force and European recommendations for PSA screening for early detection of prostate cancer [19,20]. The survey was developed with public and patient involvement and has been previously described in detail [21]. We previously reported an overall good representation of the general population of men aged 45 to 70 years in our sample; however, response rates tended to increase with older age and a location of residence that was rural and had a lower tax base, fewer people with higher education, and fewer people with non-Western backgrounds [13]. In agreement with prior research, our analysis also suggested that the response rate may have varied across different versions of the questionnaire [13,18,22-24].

The questionnaire contained items covering sociodemographic information, personality (using the validated Big Five Inventory-10 [25]), preferences for control regarding health care decision-making (using the validated Control Preferences Scale [26]), purpose-designed health-related questions, and hypothetical case vignettes concerning PSA screening for prostate cancer in men. Each scenario had an identical core structure but differed regarding the degree of patient involvement, particularly information delivery (5 levels, from “no information” to “substantiated information,” ie, fully shared decision-making with a decision aid provided as an attached PDF information sheet); the decision to have a PSA test or not; and 3 different outcomes (no prostate cancer, diagnosis of a treatable prostate cancer, and diagnosis of an eventually lethal prostate cancer) potentially posing 3 different levels of distress. Because of differences in scenario content, questionnaire variants inevitably had various lengths (ie, word count), ranging from short to long variants requiring participants to read through extensive survey material and ranging from variants with low potential for emotional distress to variants with high potential for distress. To ensure a quasi-random assignment of participants, they were sorted by date of birth and then consecutively allocated into 1 of the 30 different case vignette scenarios driving the key “exposure” variable.

In Denmark, all residents are registered in a civil registration system with a unique personal identification number. The sample for the study was identified through use of these civil registration numbers, which were obtained from the Danish Health Data Authority. Civil registration numbers permit matching to national-level statistical data on age and region and to municipal-level data from the Danish municipal statistical database [27] on the residential location of individuals, including data on rurality (ie, population density), socioeconomic surroundings (ie, municipal tax base), municipal educational level, and municipal proportion of residents with a non-Western background. The latter data are commonly used as a standard measure of state and municipality in Denmark, as well as for research purposes [28,29].

We used REDCap and distributed the survey through the digital mailbox e-Boks, which was introduced by the Danish authorities in 2001; from 2014 onward it has been a mandatory communication platform used by Danish authorities for secure communication with residents of Denmark. Residents receive an email and/or text message when they receive new mail in their private e-Boks account, which can be accessed directly with multifactor authentication to safeguard data security. e-Boks has a very high coverage of the Danish adult population, with only a small proportion of residents having actively deregistered (in 2017 this was 9.9%) [30].

To ensure a sufficient sample size, we obtained permission to invite 2 independent random samples of men aged 45 to 70 years in 2 waves, each receiving an initial invitation; a 14-day reminder sent to nonrespondents. The 2 waves were unconnected in so far as they were 2 different nonoverlapping random samples of the population. We sent out the first (winter) wave of invitations on January 24, 2019, with the 14-day reminders sent on February 7, 2019. We launched the second (spring) wave on March 7, 2019, with the 14-day reminders sent to nonrespondents on March 21, 2019.

### Statistics and Power Analysis

Power calculations and the required sample size for conducting the main study have been previously reported [31]. Categorical characteristics were reported as counts and proportions, while numerical characteristics (eg, response time) were reported as medians with IQRs or means with SDs. Odds ratios (ORs) and *P* values for predictors of response to the questionnaire were estimated by logistic regression, both for each predictor on its own and in a multivariate model adjusting for all predictors mutually. As we previously found that response rates varied with age, rurality, tax base, education, and background, we included these variables in our analysis [13]. We did not use survey weights, as our invited sample was a random sample of the total population; hence, it represented a balanced sample. The participating individuals were not perfectly representative of the population, but as our outcome was completion of the survey and not the answers provided to the survey, applying weights would have counteracted the purpose of the study, that is, to investigate patterns in who responds to a survey.

### Ethical Considerations

According to Regional Committees on Health Research Ethics for Southern Denmark assessment 2018‐1906, the project was in accordance with Danish research ethics legislation. Furthermore, use of personal data was approved by the Danish Data Protection Agency (assessment 18/31191). Informed consent for survey participation was obtained through the email invitation delivered to participants in the digital mailbox and, following pseudonymization, the data were stored in a secured environment. Participants received no compensation.

### Results

Of 24,000 potential invitees, we excluded 161 (0.67%) from the study, as at the time of the survey, they were no longer aged between 45 and 70 years. Of the 23,839 invitations sent out, 1551 (6.5%) were undeliverable because no digital mailbox was available and were marked incomplete. This number is in accordance with the expected compliance with the Danish national digital mailbox infrastructure [32]. Among the 22,288 people successfully invited through the digital mailbox, 15,532 (69.7%) provided partial or no response, and 6756 (30.3%) completed the questionnaire (Figure 1).

Following the first wave, 30.6% (3395/11,111) of respondents provided a complete response, including 20.6% (2293/11,111) before the 14-day reminder and an additional 9.9% (1102/11,111) after the reminder. Following the second wave, the complete response rate was 30.1% (3361/11,177), including 21.0% (2352/11,177) before the reminder and 9.0% (1009/11,177) after the reminder.

**Figure 1.**
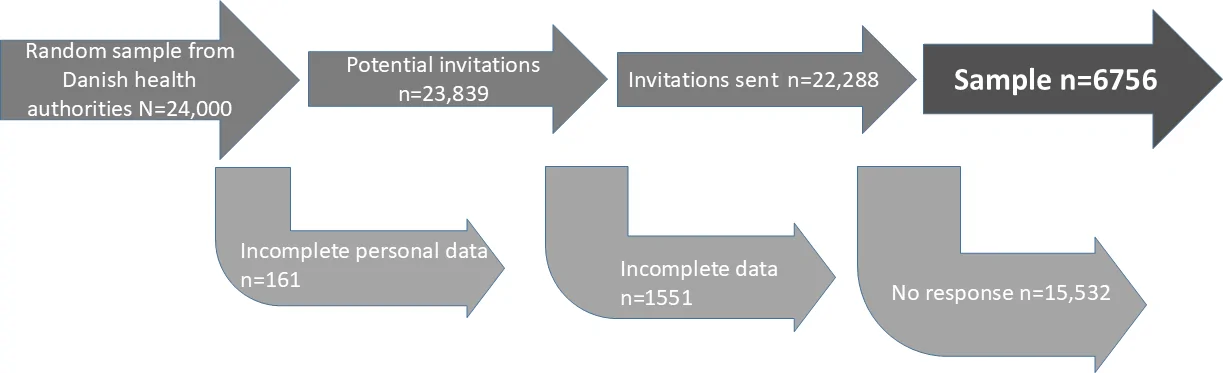
Flow chart showing retrieved sample from authorities, invitations sent out, and responses achieved constituting the sample.

Among respondents, 30% (2022/6756) responded on the day the survey was launched (Thursday), 28% (1883/6756) on the second day (Friday), 14% (948/6756) on the third day (Saturday), 10% (648/6756) on the fourth day (Sunday), 8% (567/6756) on the fifth day (Monday), 6% (387/6756) on the sixth day (Tuesday), and 4% (301/6756) on the seventh day (Wednesday), showing a gradual decrease. Most participants responded in the afternoon and early evening.

Among 1579 people who started the questionnaire but did not complete it (a number that might include people who started more than once), 1564 finished the survey homepage describing the purpose of the survey, participants’ rights, the handling of research data, and the instructions, which included pilot-testing information that the survey would take approximately 10 minutes to fill out. After this, 1477 finished the first case vignette text page, 1251 the second page, and 1149 the third page. The majority of dropouts occurred after the first questionnaire page, with only 378 remaining. Following the second questionnaire page (including variants with extra text), 177 remained; 120 remained after the third questionnaire page, 97 after the fourth questionnaire page, 79 after the fifth questionnaire page, 61 after the sixth questionnaire page; finally, 51 people remained after the seventh questionnaire page but then dropped out before finalizing the questionnaire (Figure 2).

**Figure 2.**
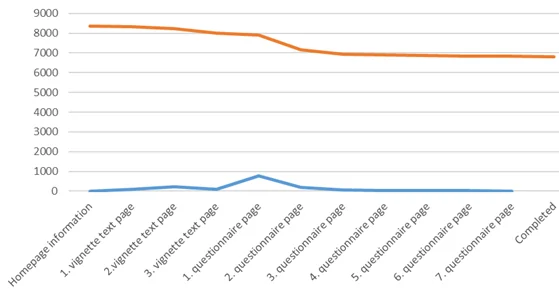
Remaining participants and number of dropouts through the questionnaire, from the homepage and to the pages of the questionnaire itself. The orange curve shows remaining participants at each stage and the blue curve shows the number of dropouts.

The average response time was 11.1 (SD 46.7) minutes and the median response time was 8.2 (IQR 6.3-10.9) minutes. The large difference between the average and median response times was because some responders needed several hours to complete the survey (Figure 3). There was some correlation between the time used to respond to the questionnaire and the number of middle-category responses, but this only reached statistical significance for questionnaire items 2 (*P*<.001) and 9 (*P*=.02). The median age of respondents was 59 (IQR 53-65) years. Other survey respondent characteristics are presented in Table 1.

**Figure 3.**
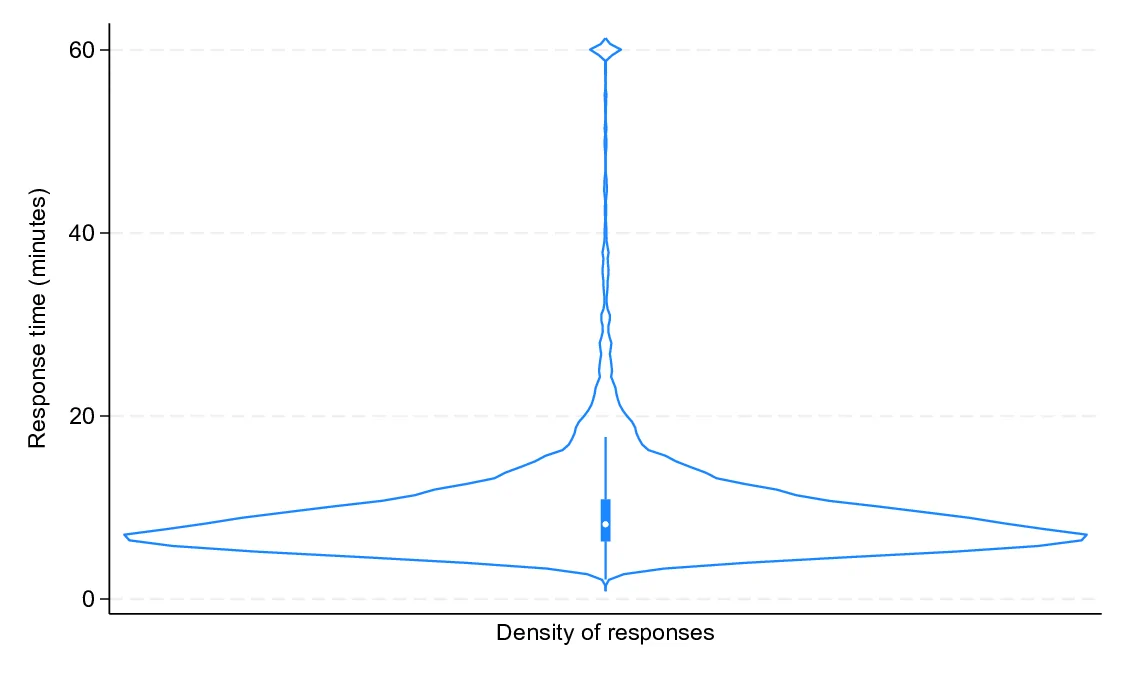
Violin plot of response times in minutes, with 33 responses of more than 60 minutes winsorized to 60 minutes to improve readability.

**Table 1.** Survey respondent characteristics and number of respondents compared to number of nonrespondents.

Respondent characteristics	Respondents (n=6756), n (% )	Nonrespondents (n=15,532), n (%)
Age groups (years)		
Youngest (45‐55)	2160 (32)	7384 (48)
Middle (55‐65)	2774 (41)	5662 (36)
Oldest (65‐70)	1822 (27)	2486 (16)
Municipal tax base per person (DKK^a^)		
Lower half (157,077‐180,414)	3516 (52)	7669 (49)
Upper half (180,437‐377,409)	3240 (48)	7863 (51)
Urban or rural area (dichotomized according to population density in persons/km2)		
Rural (15‐145)	3553 (53)	7755 (50)
Urban (147‐11,949)	3203 (47)	7777 (50)
Proportion of people in a municipality with a non-Western background		
Least half (0.8%‐3.9%)	3587 (53)	7864 (51)
Highest half (3.9%‐14.4%)	3169 (47)	7668 (49)
Proportion of people aged 25‐64 years with higher education		
Lower half (15.3%‐28.1%)	3592 (53)	7884 (51)
Upper half (28.2%‐59.8%)	3164 (47)	7648 (49)
Location (5 regions)		
Capital Region	1740 (26)	4660 (30)
Zealand	1104 (16)	2456 (16)
Southern Denmark	1611 (24)	3350 (22)
Central Region	1614 (24)	3423 (22)
Northern Jutland	687 (10)	1643 (11)

a 1 DKK=US $0.156.

Table 2 presents the associations between response rates and different survey characteristics. In addition to the target group characteristics, several features of the survey were statistically significantly associated with the survey response rate, including length, attachment of PDF information material with the survey (as a decision aid, as described above; unadjusted analysis), and emotional content (adjusted analysis).

**Table 2.** Association of survey and respondent parameters with response rates in a logistic regression (raw and adjusted analyses).

				Response rate, n (%)	Unadjusted OR^a^ (95% CI)	*P* value	Adjusted OR (95% CI)	*P* value
Survey characteristics								
	Questionnaire length (words)							
		900‐1000 (level 1)		33 (727/2223)	1 (reference)		1 (reference)	
		1000‐1100 (level 2)		30 (1799/5963)	0.89 (0.80‐0.99)	.03	0.90 (0.81-1.00)	.048
		1100‐1200 (level 3)		31 (1614/5173)	0.93 (0.84‐1.04)	.20	0.96 (0.85‐1.09)	.53
		1200‐1300 (level 4)		30 (1320/4438)	0.87 (0.78‐0.97)	.01	0.99 (0.84‐1.18)	.95
		2000‐2100 (level 5)		30 (892/3001)	0.87 (0.77‐0.98)	.02	0.87 (0.77‐0.98)	.02
		2100‐2200 (level 6)		27 (404/1490)	0.77 (0.66‐0.88)	<.001	0.87 (0.72‐1.06)	.17
	Material attached or not							
		No attachment		31 (5460/17,797)	1 (reference)	—^b^	N/A^c^	N/A
		With attachment		29 (1296/4491)	0.92 (0.85‐0.98)	.02	N/A	N/A
	Content (3 levels of emotional distress)							
		No		31 (2308/7467)	1 (reference)	—	1 (reference)	—
		Little		31 (2304/7406)	1.01 (0.94‐1.08)	.79	0.98 (0.89‐1.08)	.70
		Much		29 (2144/7415)	0.91 (0.85‐0.98)	.008	0.86 (0.75‐0.99)	.03
Survey distribution characteristics								
	Timing of survey invitation							
		Winter wave (January 2019)		31 (3395/11,111)	1 (reference)	—	1 (reference)	—
		Spring wave (March 2019)		30 (3361/11,177)	0.98 (0.92‐1.03)	0.43	0.98 (0.93‐1.04)	.58
Characteristics of survey target group								
		Age (years)						
			Youngest (45-55)	23 (2160/9544)	1 (reference)	—	1 (reference)	—
			Middle (55-65)	33 (2774/8436)	1.67 (1.57‐1.79)	<.001	1.67 (1.56‐1.78)	<.001
			Oldest (65-70)	42 (1822/4308)	2.51 (2.32‐2.71)	<.001	2.49 (2.31‐2.70)	<.001
		Municipal tax base per resident						
			Lower half	31 (3516/11,185)	1 (reference)	—	1 (reference)	—
			Upper half	29 (3240/11,103)	0.90 (0.85‐0.95)	<.001	1.01 (0.92‐1.11)	.83
		Urban or rural area (dichotomized according to population density)						
			Rural (lower populated half)	31 (3553/11,308)	1 (reference)	—	1 (reference)	—
			Urban (upper populated half)	29 (3203/10,980)	0.90 (0.85‐0.95)	<.001	1.08 (0.99‐1.19)	.10
		Proportion of residents with a non-Western background in municipality						
			Lower half	31 (3587/11,451)	1 (reference)	—	1 (reference)	—
			Upper half	29 (3169/10,837)	0.91 (0.86‐0.96)	.001	0.95 (0.87‐1.03)	.20
		Proportion of residents aged 25‐64 y with higher education						
			Lower half	31 (3592/11,476)	1 (reference)	—	1 (reference)	—
			Upper half	29 (3164/10,812)	0.91 (0.86‐0.96)	.001	0.95 (0.88‐1.03)	.24
		Geographical location						
			Capital Region	27 (1740/6400)	1 (reference)	—	1 (reference)	—
			Zealand	31 (1104/3560)	1.20 (1.10‐1.32)	<.001	1.05 (0.92‐1.20)	.48
			Southern Denmark	32 (1611/4961)	1.29 (1.19‐1.40)	<.001	1.24 (1.13‐1.37)	<.001
			Central Region	32 (1614/5037)	1.26 (1.16‐1.37)	<.001	1.29 (1.16‐1.43)	<.001
			Northern Jutland	29 (687/2330)	1.12 (1.01‐1.24)	.03	1.16 (1.04‐1.29)	.007

a OR: odds ratio.

b Not applicable.

c Not included in adjusted analysis due to full collinearity with questionnaire length.

## Discussion

### Summary of Main Study Findings

This study examined questionnaire characteristics that predicted participation in an internet-based survey that used a national digital mailbox; the results showed that reminders added another half to the response rate, while longer questionnaire variants, questionnaires with attached informational material, and questionnaires with potentially distressful emotional content tended to yield lower response rates. Below, we discuss these findings in the context of the existing literature.

### Study Findings and Comparison to Existing Literature

### Questionnaire Length and Content

We found that the response rate was inversely associated with the length of the questionnaire. Among those who started completing the questionnaire but gave up, the majority stopped immediately after switching from the first introductory text page to the first questionnaire item requiring a response. After this point, the dropout rates were greatest for the earlier items. Many dropouts, however, also occurred during the part of the questionnaire with vignette variants that had more text material. Corresponding with this, various previous studies have found a relationship between increasing questionnaire length and decreasing response rates in both mail and internet surveys [18,22-24], a relationship that was also established in a Cochrane review of postal and electronic surveys [8]. The relationship between questionnaire length and response rate seems to hold whether looking at the number of survey pages, screens, or items or at the time to complete the questionnaire [18]. In our survey, the average response time was 11 minutes, while some previous research has suggested that a completion time of no more than 13 minutes is necessary to obtain a good response rate [18]. Likewise, our finding that questionnaire variants with more potential to cause distress had statistically significantly lower response rates confirms findings from the Cochrane review [8] and a widely held belief that questions causing emotional discomfort decrease participation [18,33].

### Geographical and Target Group Factors

We found that older men had higher response rates than younger men. On the other hand, people residing in areas with a higher tax base, areas with a higher proportion of residents with higher education, and areas with more residents with a non-Western background had lower response rates than people living in lower tax-base areas, areas with fewer people with higher education, and areas with fewer people with a non-Western background. In addition, response rates were lower in urban areas than in rural areas. There has been a paucity of research on the role of geographical factors in survey response rates [18]. However, in a Norwegian study using data from a national survey of patient experiences with maternity care, Sjetne et al [16] found that the likelihood of responding to surveys was strongly influenced by geographical location. In our study, we found that response rates were lowest in the Danish Capital Region. Corresponding with this, in addition to an overall decline in survey participation rates, Tolonen and colleagues [12] found an overall lower participation rate in the capital region. Regarding target group characteristics affecting response rates, Sjetne et al [16] found that the likelihood of responding to a survey was strongly influenced by background variables, including age. Hence, older age was associated with higher response probability. Likewise, in a previous national study conducted with women in the United Kingdom, respondents were more likely to be older, married, living in the least-deprived areas, and to be born in the United Kingdom [17]. Hence, our findings suggest that older people may not necessarily be less inclined to participate in research making use of web surveys and digital platforms. Findings of higher participation among older people thereby contradict previous digital health technology evaluation studies highlighting nonparticipation by older people and those with less experience with digital platforms [34].

### Effect of Reminders

Previous studies have found large variation in the effect of reminders [18,35]. Some variation may, however, be explained by differences between paper-based and internet surveys. For example, Cantuaria and Blanes-Vidal [35] compared survey responses collected with mail and internet methods and found that reminders had a stronger effect on increasing internet survey response rates than mail response rates. Their study was relatively small; they initially invited all participants through mail, finding that 2 reminders added a little more than half to the response rate in the mail survey and almost doubled the number of internet responses [35]. We made no such comparison, as we invited all participants via the Danish digital mailbox and only used 1 reminder; moreover, our reminder did not achieve the same effectiveness as the one sent to internet participants by Cantuaria and Blanes-Vidal [35]. In our study, there was a 31% response rate following the first wave, including 21% before the 14-day reminder and an additional 10% after the reminder. Likewise, in the second wave, the reminder added roughly 50% to the response rate, suggesting a rule of thumb that reminders may add another half to the response rate achieved by an initial invitation. Previous survey studies have suggested comparable effects of reminders. For example, a Danish national self-administered health survey found that cumulative response rates after first, second, and third mailings were 36.7%, 50.3% and 59.5%, respectively [14]. A recent Danish national self-administered health survey found that the cumulative response proportions were 19.1%, 28.9%, and 37.2%, simultaneously pointing to a tendency toward declining participation in public health surveys [7]. Even if it did not investigate authorities’ digital mailbox solutions as a mode of survey distribution, a Cochrane review of methods to increase the response to postal and electronic questionnaires found a similar pattern, and findings from previous studies suggest that our rule of thumb may extend to additional reminders, a way of increasing response rates often used in survey research [8]. In parallel, ethical considerations related to respect for people’s right to prioritize their time and resources might speak in favor of limiting the use of reminders.

The Cochrane review found that, for postal questionnaire studies, the odds of response significantly increased when using a special (for example registered or certified) delivery service [8]. Thus, using modes of invitations signaling authority and quality may be beneficial in terms of promoting participation; however, this again gives rise to ethical considerations. For example, exposing large numbers of people to surveys causing emotional distress or exposing people to low-quality questionnaire studies that take advantage of platforms established by authorities to communicate important information may undermine people’s trust in the platform. The “quality stamp” only works if its credibility is not watered down through overuse by researchers and by people starting to perceive the platform as irrelevant. An overall response rate just exceeding 30% to a public health survey benefiting from the web and platform opportunities described in our study is not high. Our findings should be seen in the context of emerging evidence that participation in web-based, health-related surveys is decreasing [5-7]. Among the possible causes of decreasing participation are, again, survey fatigue, with the increasing number of surveys being sent out making people less likely to respond to any single request [3-7].

### Limitations

A limitation of this study was that it only included men aged 45 to 70. Response rates have previously been found to be greater among women than men [5,36]. Another limitation was that the study was conducted using data from 2019; it could be claimed that much has changed in the digital world since then. Still, electronic surveys are widely used for research, and digital mailboxes are an increasingly used channel for distributing electronic surveys in countries with such platforms. Moreover, we did not test whether people who responded after the reminder differed from those who responded before in terms of, for example, response patterns.

It can be questioned whether findings on participation and response rates to surveys distributed via a mandatory, high-population coverage, authority-provided digital mailbox can be generalized, or to what extent, to voluntary or low-coverage mailboxes. High-coverage and mandatory systems may tend to decrease coverage bias, while voluntary or low-coverage systems may suffer to a higher extent from nonresponse bias and self-selection errors. Additionally, in mandatory systems, individuals are legally required to register and check their mailboxes. As has been discussed above, when a survey is distributed through a mandatory system, recipients may associate it with official or legal duties, driving up response rates. Contrarily, voluntary platforms lack this legal obligation and authoritative weight. Moreover, in mandatory systems with almost complete population coverage, the sampling frame matches the target population, while in voluntary or low-coverage systems, certain demographics (eg, older people, low-income individuals, and those with lower digital literacy) may be structurally excluded or less likely to opt in, thereby skewing results. Finally, in voluntary systems, respondents who choose to engage may have stronger preexisting opinions on the survey topic than nonrespondents. Mandatory systems may thus capture a broader spectrum of the public, including indifferent individuals. Thus, the findings of the present study may be generalizable to other mandatory or high-coverage systems, whereas estimating the general population’s behavior in areas with voluntary or lower-coverage digital mailbox solutions may to some extent require statistical correction. The latter can be done, for example, through use of propensity score matching (thereby modeling the probability of an individual responding through use of administrative data, followed by reweighting of the sample to match nonparticipants) or through adjustment of data using known population benchmarks (eg, age, education, and income level) to correct for misrepresented groups.

As indicated in the Methods section, the assignment procedure was based on date-of-birth sorting rather than truly random sorting, representing a potential limitation.

Finally, the study only investigated a limited number of factors that can affect survey participation and did not examine factors such as question wording (eg, keeping questions simple), display type (eg, graphics and progress indicators), use of incentives (eg, gifts), or whether the survey is specifically aimed at the invited group (eg, a survey about an illness sent to people with that illness) [6,8,18].

### Conclusion

Online surveys are now routinely used for gathering data in social research, public health, and related disciplines, and in some countries, surveys increasingly use web platforms provided by national authorities. However, nonparticipation remains a problem that can bias findings. In our study, survey characteristics such as length, file attachment, and emotionally distressful content were associated with lower response rates. Our study therefore largely confirms what is already known from predigital survey research and online research using other modes of survey distribution. Existing patterns seemingly hold in a national digital mailbox setting, thereby emphasizing that traditional principles regarding survey design and factors affecting survey participation also apply to newer technologies. This underscores the importance of considering these factors when conducting internet-based surveys distributed through digital mailboxes. Hence, questionnaires should be kept as short as possible, and the estimated completion time should be communicated honestly. Response rates can also still be considerably increased through reminders. Nonetheless, participation in public health surveys continues to decline, and despite easier procedures, it is therefore more important than ever that researchers, before launching a new survey, evaluate if the required information is already available through, for example, existing datasets, or whether it can be obtained through other means.

## Supplementary material

10.2196/89761 Checklist 1 STROBE checklist.

## Abbreviations

OR: odds ratio
PSA: prostate-specific antigen
