# Supplementary material for: Factors Influencing Response Rates in National Internet-Based Questionnaires: Analysis of a Large National Survey
Source: JMIR Public Health Surveill. 2026 Sep 30;12:e89761. doi: 10.2196/89761 (PMC13626076; doi:10.2196/89761)
Supplement: Supplementary file 1 [file publichealth-v12-e89761-s001.pdf]

Factors Influencing Response Rates in National Internet-Based Questionnaires: Analysis of a Large National Survey

STROBE Statement—Checklist of items that should be included in reports of *cross-sectional studies*

	Item No	Recommendation	
**Title and abstract**	1	(*a*) Indicate the study's design with a commonly used term in the title or the abstract	Please see Title
		(*b*) Provide in the abstract an informative and balanced summary of what was done and what was found	Please see abstract, lines 1-19
**Introduction**			
Background/rationale	2	Explain the scientific background and rationale for the investigation being reported	Please see introduction, lines 1-48
Objectives	3	State specific objectives, including any prespecified hypotheses	Please see introduction, lines 49-52
**Methods**			
Study design	4	Present key elements of study design early in the paper	Please see methods, first para
Setting	5	Describe the setting, locations, and relevant dates, including periods of recruitment, exposure, follow-up, and data collection	Please see methods, first para
Participants	6	(*a*) Give the eligibility criteria, and the sources and methods of selection of participants	Please see methods, first para
Variables	7	Clearly define all outcomes, exposures, predictors, potential confounders, and effect modifiers. Give diagnostic criteria, if applicable	Please see methods, first, second and third paras
Data sources/ measurement	8*	For each variable of interest, give sources of data and details of methods of assessment (measurement). Describe comparability of assessment methods if there is more than one group	Please see methods, first, second and third paras
Bias	9	Describe any efforts to address potential sources of bias	Please see methods, second para
Study size	10	Explain how the study size was arrived at	Please see 'Statistics and Power Analysis'
Quantitative variables	11	Explain how quantitative variables were handled in the analyses. If applicable, describe which groupings were chosen and why	Please see methods, first, second and third paras
Statistical methods	12	(*a*) Describe all statistical methods, including those used to control for confounding	Please see 'Statistics and Power Analysis'
		(*b*) Describe any methods used to examine subgroups and interactions	
		(*c*) Explain how missing data were addressed	
		(*d*) If applicable, describe analytical methods taking account of sampling strategy	
		(*e*) Describe any sensitivity analyses	
**Results**			
Participants	13*	(a) Report numbers of individuals at each stage of study—eg numbers potentially eligible, examined for eligibility, confirmed eligible, included in the study, completing follow-up, and analysed	Please see Figure 1
		(b) Give reasons for non-participation at each stage	Please see Figure 1
		(c) Consider use of a flow diagram	Please see Figure 1
Descriptive data	14*	(a) Give characteristics of study participants (eg demographic, clinical, social) and information on exposures and potential confounders	Please see table 1
		(b) Indicate number of participants with missing data for each variable of interest	NA
Outcome data	15*	Report numbers of outcome events or summary measures	Please see table 1
Main results	16	(*a*) Give unadjusted estimates and, if applicable, confounder-adjusted estimates and their precision (eg, 95% confidence interval). Make clear which confounders were adjusted for and why they were included	Please see table 2
		(*b*) Report category boundaries when continuous variables were categorized	Please see table 2
		(*c*) If relevant, consider translating estimates of relative risk into absolute risk for a meaningful time period	NA
Other analyses	17	Report other analyses done—eg analyses of subgroups and interactions, and sensitivity analyses	Please see Results, paras 1-5

**Discussion**			
Key results	18	Summarise key results with reference to study objectives	Please see Discussion, 'Summary of Main Study Findings'
Limitations	19	Discuss limitations of the study, taking into account sources of potential bias or imprecision. Discuss both direction and magnitude of any potential bias	Please see discussion, 'Limitations'
Interpretation	20	Give a cautious overall interpretation of results considering objectives, limitations, multiplicity of analyses, results from similar studies, and other relevant evidence	Please see discussion, paras 1-4
Generalisability	21	Discuss the generalisability (external validity) of the study results	Please see discussion, 'Limitations'
**Other information**			
Funding	22	Give the source of funding and the role of the funders for the present study and, if applicable, for the original study on which the present article is based	Please see separate funding statement

*Give information separately for exposed and unexposed groups.

Note: An Explanation and Elaboration article discusses each checklist item and gives methodological background and published examples of transparent reporting. The STROBE checklist is best used in conjunction with this article (freely available on the Web sites of PLoS Medicine at http://www.plosmedicine.org/, Annals of Internal Medicine at http://www.annals.org/, and Epidemiology at http://www.epidem.com/). Information on the STROBE Initiative is available at www.strobe-statement.org.
